# The Sequential-Weight Illusion

**DOI:** 10.1177/2041669518790275

**Published:** 2018-07-25

**Authors:** Guido Maiello, Vivian C. Paulun, Lina K. Klein, Roland W. Fleming

**Affiliations:** Department of Experimental Psychology, University of Giessen, Germany

**Keywords:** weight perception, sensorimotor memory, grasping, material perception, haptics/touch, perception/action, reaching/grasping, visuo-haptic interactions

## Abstract

We report an illusion in which the felt weight of an object changes depending on whether a previously manipulated object was lighter or heavier. The illusion is not modulated by visual weight cues, yet it transfers across hands.

While constructing stimuli for an experiment, we noticed a curious phenomenon. If we picked up an object made of brass, then a lighter object made of wood, then the brass object again, the brass object felt noticeably heavier the second time. The illusion was striking because it occurred in spite of our strong expectation that weight should remain stable across time.

At our lab’s weekly meeting, we ran an informal experiment to demonstrate and discuss this curious effect. We created two 12.5 × 2.5 × 2.5 cm objects, one of wood (50 g) and another of brass (670 g). We wrapped both in paper, rendering them visually indistinguishable, and placed them in a container. Our colleagues were asked to hold out their right hand palm up. First, we placed the heavy object on their palm. After returning it to the container, we put the light object on the participant’s palm. We then placed the light object in the container and once more placed the heavy object on the participant’s palm. Finally, the heavy object was returned to the container, and the participant asked to rate the weight of the three objects, not knowing that the same heavy object had been presented twice. Participants wrote their weight ratings on paper slips. Data analysis was conducted using pen and paper (Figure 1). Unsurprisingly, the second object was rated much lighter than first and third objects. Critically, however, nearly all participants reported the third object to be heavier than the first, despite their being physically identical. Only 1/10 participants veridically reported perceiving first and third objects as weighing the same; 4/10 participants were not naïve, as we had demonstrated the effect to them in the days prior to the meeting. Both naïve and nonnaïve participants experienced the illusory weight change; knowledge of the illusion did not appear to weaken the effect, suggesting it might be cognitively impenetrable (Lupyan, 2015).

**Figure 1.**
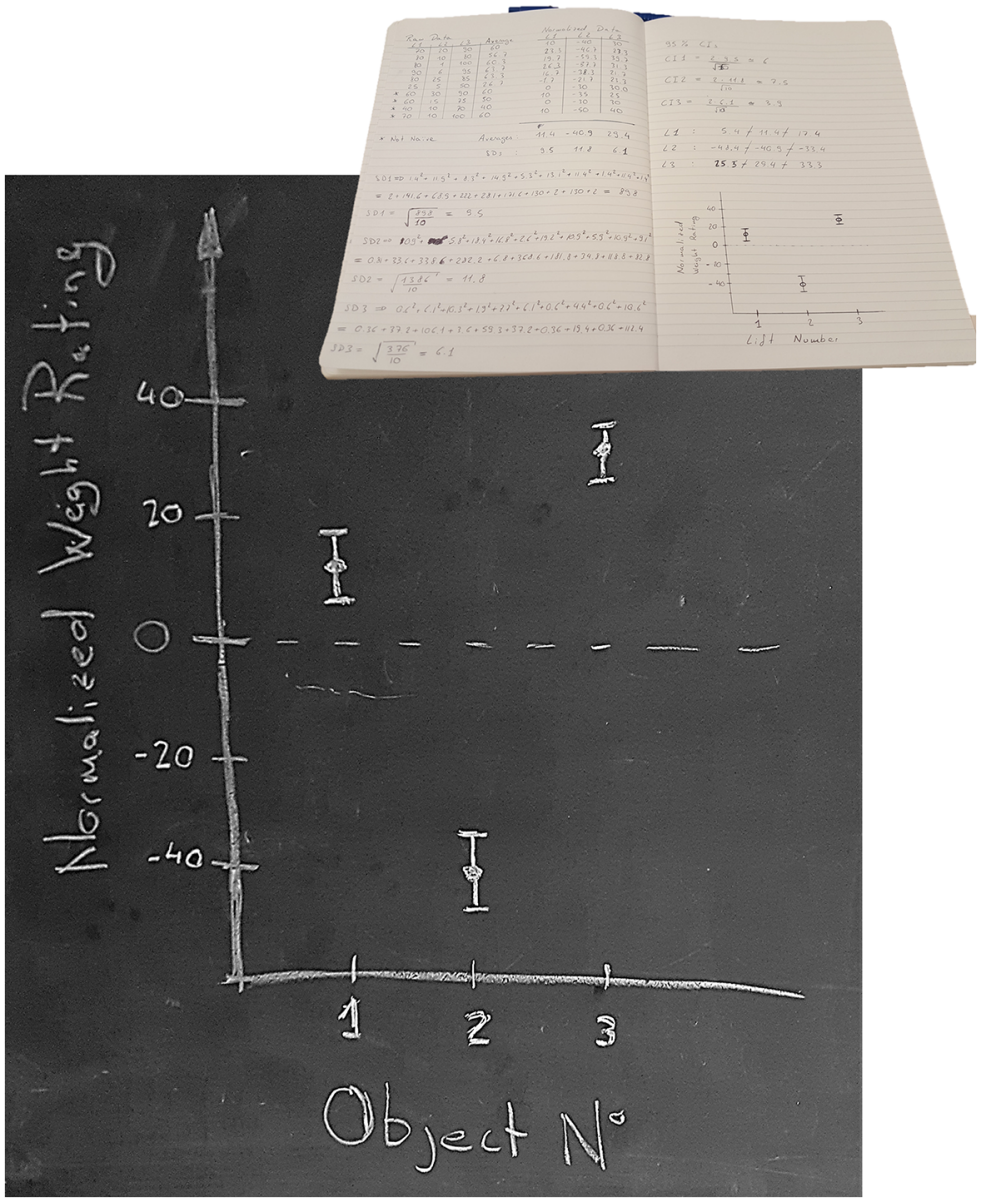
Very formal results from the informal experiment. Data and analyses are openly shared directly from the experimenter’s lab notebook. Rating data were normalized by subtracting each participant’s mean rating. Mean normalized weight ratings were hand-plotted as a function of object number onto the conference room black board. Error bars are 95% confidence intervals. Objects 1 and 3 are the same.

Following the discussions that took place at the group meeting, we concluded that the illusion is likely related to the interaction between short-term motor adaptation and violations of sensorimotor expectations (van Polanen & Davare, 2015). The forces that humans use to pick up an object tend to be scaled based on previous lifts. The light wooden object therefore likely induced participants to use less force to hold the heavy object the second time around, which resulted in the heavy object feeling heavier. We set out to test whether this effect would survive cognition, that is, knowledge of the relationships between the weights of the objects.

We recruited 70 participants: 30 for Experiment 1 (19 women, 1 left-handed, mean ± *SD* age: 27 ± 7), 20 for Experiment 2 (9 women, 2 left-handed, mean ± *SD* age: 29 ± 7), and 20 for Experiment 3 (16 women, 0 left-handed, mean ± *SD* age: 25 ± 4). Participants were staff and students from the University of Giessen and provided written informed consent. Procedures were approved by the local ethics committee of the University of Giessen and adhered to the tenets of the Declaration of Helsinki.

Figure 2 shows four rectangular cuboids of dimensions 12.5 × 2.5 × 2.5 cm employed as stimuli. In all experiments, participants performed two sessions at least 10 minutes apart. The task was the same in both sessions. Participants stood in a naturally lit room with two stimuli ca. 20 cm in front of them, a target object (T) and a bias object (B). Participants were asked to lift and place back down object T, then B, and then T again. Participants then rated, on a 1 to 100 scale, how heavy T felt on the first and third lifts. Participants were explicitly told not to rate B. Each session, participants performed one single sequence of three lifts. Data were recorded using a simple data entry program written in MATLAB R2016b (MathWorks).

**Figure 2.**
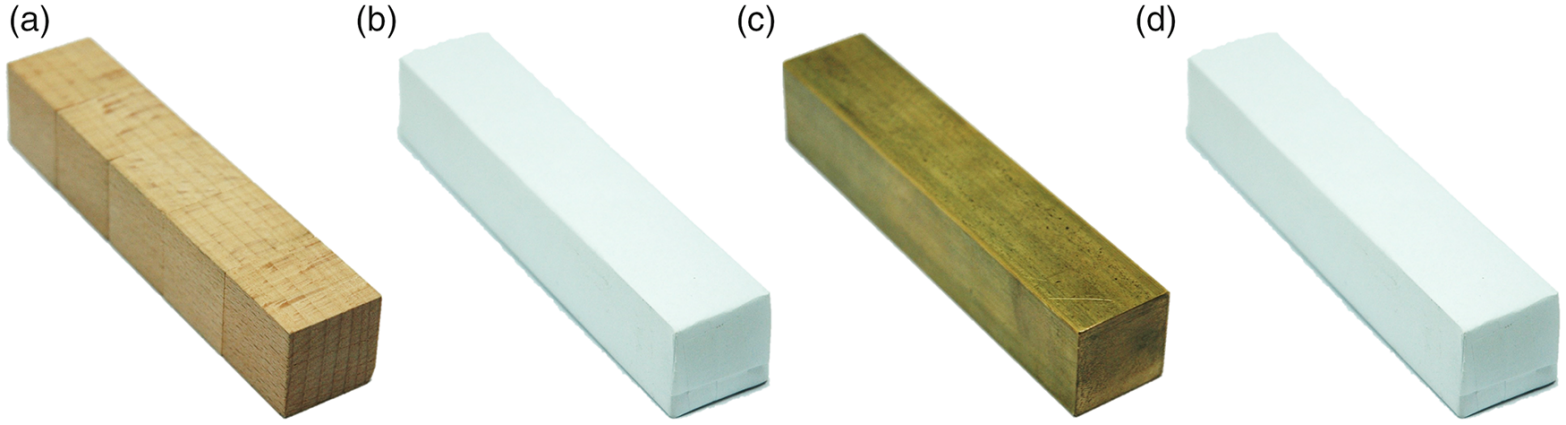
Stimuli. Four objects, two (a and b) of beech wood and two (c and d) of solid brass. (a and c) Valid and (b and d) invalid visual cues to weight.

Experiment 1 tested whether the perception of light-weight objects would be biased by lifting a heavy object. The material-weight illusion (Wolfe, 1898) demonstrates that our brain combines visual and sensorimotor weight estimates when making weight judgments. Therefore, we also tested whether visual cues to weight would modulate sensorimotor biasing of weight perception. T was made of wood and B of brass. In a valid visual cue session (V+), participants could see the materials. In an invalid visual cue session (V−), participants were presented with the objects wrapped in paper. To control for session order, half the participants performed V+ first and half performed V− first. All lifts were performed with the right hand. Ratings were analyzed using a 2 (first vs. third lift) × 2 (V+ vs. V−) within-subject analysis of variance (ANOVA).

Figure 3(a) shows that the wooden object felt 40% lighter (median percent weight change) after lifting the brass object, *F*(1, 29) = 20.31, *p* = .00010. Visual cues did not affect perceived weight, *F*(1, 29) = 0.65, *p* = .43, nor did they modulate the illusory decrease in perceived weight, *F*(1, 29) = 0.37, *p* = .55.

**Figure 3.**
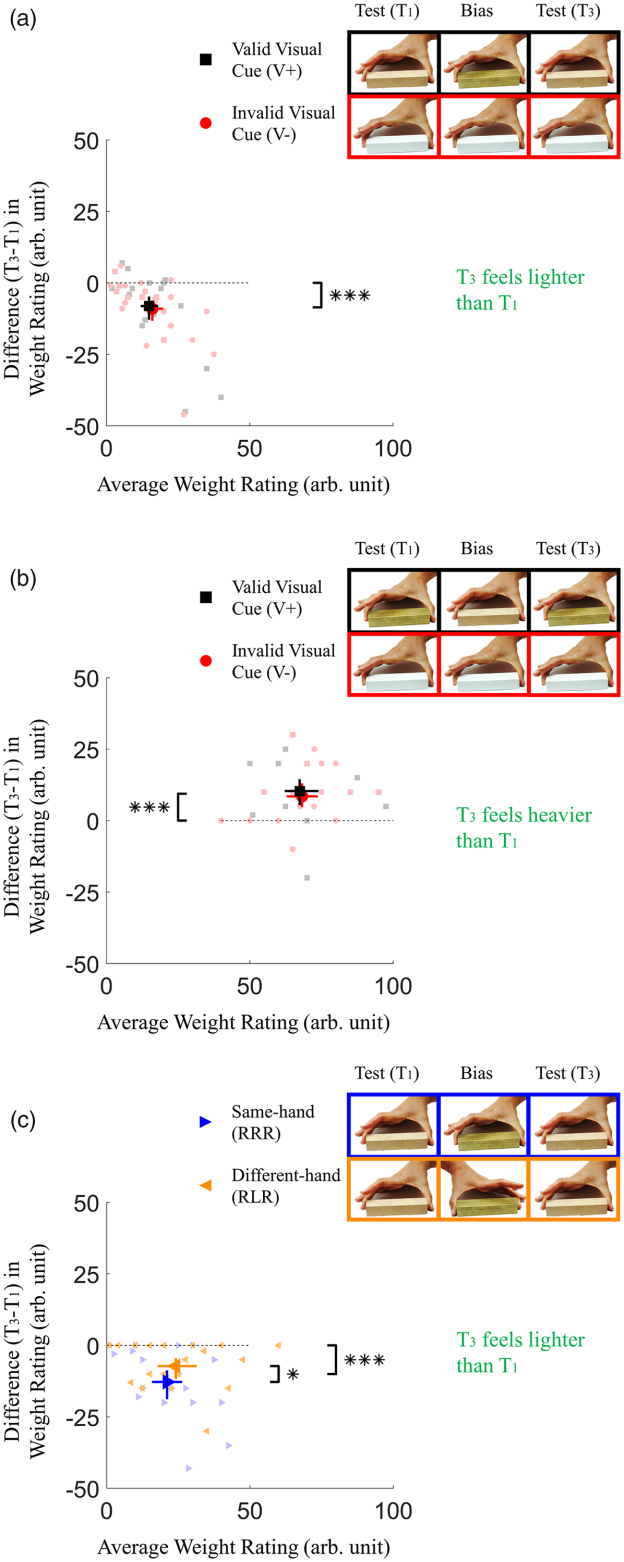
Illusory shifts in perceived weight. (a) Experiment 1. (b) Experiment 2. (c) Experiment 3. In all panels, the difference in weight rating between third and first lifts (which compactly summarizes the first vs. third lift ANOVA main effect) is plotted as a function of the mean rating across third and first lifts. Small markers are individual subject data, large markers are group means, error bars are 95% bootstrapped confidence intervals. *p < .05. ***p < .001.

van Polanen and Davare (2015) reported that sensorimotor biases occur with light but not heavy objects, yet our informal pilot suggested the illusion also worked with heavy objects. Thus, in Experiment 2, we tested whether the perception of heavy objects would be biased by lifting lighter objects, and again whether visual cues modulated this effect. Thus, T was brass and B was wood. V+ and V− sessions were as in Experiment 1, with half the participants performing V+ first and half V− first.

Figure 3(b) shows that the brass object felt 13% heavier after having lifted the wooden object, *F*(1, 19) = 20.27, *p* = .00024. Again, visual weight cues had no effect, *F*(1, 19) = 0.07, *p* = .79, nor did they modulate the main effect, *F*(1, 19) = 0.71, *p* = .41.

Chang, Flanagan, and Goodale (2008) showed that intermanual transfer of force control is not influenced by perceived weight. In Experiment 3, we tested whether sensorimotor biasing of weight perception would transfer across hands. T was wood and B was brass. Participants were presented with unwrapped objects. In a same-hand session (RRR), participants performed all three lifts with their right hand. In a different-hand session (RLR), participants lifted T with their right hand and B with their left hand. Half the participants performed RRR first and half did RLR first. Ratings were analyzed using a 2 (first vs. third lift) × 2 (RRR vs. RLR) within-subject ANOVA.

We found that lifting a heavy object with one hand biased the felt weight of a light object lifted with the other hand. Figure 3(c) shows that T felt on average 33% lighter after lifting B, *F*(1, 19) = 29.67, *p* = .000030. Ratings were similar in the RRR and RLR sessions, *F*(1, 19) = 2.96, *p* = .10. However, lifting B with the other hand than T provided a 13% decrease in perceived weight, a significantly smaller illusory weight shift than the 46% decrease in perceived weight occurring when executing all lifts with the same hand, *F*(1, 19) = 4.48, *p* = .048.

In conclusion, we demonstrate a curious phenomenon in which the haptically perceived weight of an object can change in front our eyes. Contrary to the material-weight illusion, we find that haptic weight perception is not modulated by visual cues to weight. We also find that sensorimotor memory can transfer, at least in part, across hands. These illusory shifts in perceived weight challenge our strong expectations that object weight should remain constant throughout time. Our findings highlight the importance of considering trial order effects in experiments on haptic weight perception, as the felt weight on any given trial will be influenced by the sequence of previous trials.

## Data Availability

Data and analysis scripts are available from the Zenodo database (doi:10.5281/zenodo.1246130).

## References

[bibr1-2041669518790275] Chang E. C. Flanagan J. R. Goodale M. A. (2008) The intermanual transfer of anticipatory force control in precision grip lifting is not influenced by the perception of weight. Experimental Brain Research 185: 319–329. 17934725 10.1007/s00221-007-1156-0

[bibr2-2041669518790275] Lupyan G. (2015) Cognitive penetrability of perception in the age of prediction: Predictive systems are penetrable systems. Review of Philosophy and Psychology 6: 547–569.

[bibr3-2041669518790275] van Polanen V. Davare M. (2015) Sensorimotor memory biases weight perception during object lifting. Frontiers in Human Neuroscience 9: 700. 26778993 10.3389/fnhum.2015.00700 PMC4689183

[bibr4-2041669518790275] Wolfe H. K. (1898) Some effects of size on judgments of weight. Psychological Review 5: 25.

